# Preparedness to prescribe antibiotics responsibly: a comparison between final year medical students in France and Sweden

**DOI:** 10.1007/s10096-019-03494-2

**Published:** 2019-02-15

**Authors:** Oliver James Dyar, Maria Lund, Cecilia Lindsjö, Cecilia Stålsby Lundborg, Céline Pulcini, on behalf of the French-Swedish Student-PREPARE ESGAP working group

**Affiliations:** 1 Department of Public Health Sciences, Karolinska Institutet, Stockholm, Sweden; 2 Université de Lorraine, APEMAC, F-54000 Nancy, France; 3 Université de Lorraine, CHRU-Nancy, Infectious Diseases Department, F-54000 Nancy, France

**Keywords:** Antimicrobial stewardship, Education, Training, Prudent prescribing, Questionnaire

## Abstract

Students should graduate from medical school feeling prepared to prescribe antibiotics responsibly. We assessed self-reported preparedness among students at medical schools in Europe, and we focus here on the results from students in Sweden and France, countries with wide differences in the intensity of antibiotic consumption and burden of antibiotic resistance. We conducted a cross-sectional web-based survey in 2015, based on a comprehensive set of topics related to prudent antibiotic use. All final year students at a medical school in France or Sweden were eligible to participate. Preparedness scores were calculated for each student, and mean scores were compared at medical school and country levels. Comparisons were also made on availability of teaching methods. We received responses from 2085/7653 (response rate 27.2%) students from 31/34 eligible medical schools in France and 302/1124 (26.9%) students from 7/7 schools in Sweden. The relative ranking order of curriculum topics by preparedness level was consistent between countries, but students in Sweden had higher self-reported levels of preparedness in 21/27 topics. There was higher availability for eight of nine teaching methods at Swedish medical schools. Students in France were more likely to report a need for further education on antibiotic use (63.5% vs. 20.3%, *p* < 0.001). Final year students in France report lower levels of preparedness, less availability of teaching methods and higher needs for more education on antibiotic use. Furthermore, we have identified specific areas for improvement in education on prudent antibiotic use in both Sweden and France.

**Electronic supplementary material:**

The online version of this article (10.1007/s10096-019-03494-2) contains supplementary material, which is available to authorized users.

## Introduction

During their undergraduate training, medical students must learn the fundamentals of infection diagnosis and management, and the connections between antibiotic use and antibiotic resistance [1, 2]. Furthermore, they should be prepared to prescribe antimicrobials responsibly when they commence work as junior doctors [3, 4].

Final year medical students in 28 European countries were invited in 2015 to participate in Student-PREPARE, a study assessing self-reported preparedness in a comprehensive set of topics related to responsible antibiotic use. The European-level results have been published [5]. This manuscript presents a detailed sub-group analysis of responses from all participating medical schools in France and Sweden, which were selected for further analyses for two reasons: first, there are significant differences between these countries in the intensity and type of antibiotic use [6, 7]. The consumption of antibiotics is about two times higher per capita in France based on Defined Daily Doses, (although dosing is lower in Sweden), and narrow-spectrum antibiotics are used more frequently in Sweden [8]. Second, the response rates in the study were broadly consistent for participating schools in both countries.

### Medical education in Sweden and France

In France (67 million inhabitants), 34 universities offer undergraduate medical education and the duration is 6 years. The content of the medical curricula is regulated nationally by the Ministries of Health and Higher Education. The first year offers a common core curriculum for all students who pursue medicine, pharmacy, dentistry, midwifery, and additional specific education for each discipline. The first 2 years are mostly pre-clinical, including basic sciences, and the last 4 years are mostly clinical, including clinical rotations which account for half of the final 3 years. The national curriculum is organised around clinical specialities. There is no separate period of internship in France; graduates move directly on to a postgraduate medical education period with the choice of specialty based on a national ranking examination [9]. This lasts 3 to 5 years, after which doctors obtain a specialist diploma.

In Sweden (10 million inhabitants), 7 universities offer undergraduate medical education and the duration is five and a half years. The content is not nationally regulated, but the quality of curricula is controlled and accredited by the Swedish Higher Education Authority. The structure of the education varies between medical schools; however, it is integrated around either organ systems or physiological and pathophysiological processes, or is organised around basic medical sciences in conjunction with clinical specialities. Undergraduate medical education is followed by a mandatory internship for 18–24 months before a licence to practice medicine is granted. This is followed by a postgraduate medical education period of 5 to 7 years to obtain a specialist diploma [10].

## Materials and methods

### Study design and participants

A cross-sectional multicentre online survey was conducted at medical schools in 28 European countries in 2015 [5]. All final year students at medical schools in Sweden and France could participate. Country coordinators (authors CP in France, CL and CSL in Sweden) invited all medical schools to participate. Eligible students at participating schools were sent an invitation email by a local coordinator and reminders after 2–4 and 8–14 weeks. Country coordinators could send additional reminders to schools with low response rates. The self-administered survey was accessible on SurveyMonkey®.

### Survey development

The survey tool (see Online Resource 1) was developed by a committee of international experts on antibiotic stewardship and was informed by previous studies on undergraduate curricula and among medical students [11–13]. The survey consisted of 47 items, including questions on socio-demographics, self-reported preparedness on 27 curriculum topics on prudent antibiotic use (using a 7-point Likert-type scale [5]), availability and usefulness of teaching methods, and perceived need for further education at medical school. The questionnaire was developed in English and pilot-tested with eight students in France and the UK.

### Statistical analyses

Data was exported from SurveyMonkey® and analyses were performed in Microsoft Excel® Version 2016. Responses were excluded if fewer than half of the questions of perceived preparedness were completed. Responses for preparedness on topics were condensed into two categories (1–3, insufficiently prepared; 4–7, at least sufficiently prepared). As previously described [5], “topic preparedness scores” were created at the medical school level, then the country level. These represent the percentage of students at a medical school who felt at least sufficiently prepared on each topic. Separately, “global preparedness” scores were created for each student, then aggregated at the medical school level, and country level. These represent the proportion of all 27 topics in which a student felt at least sufficiently prepared.

Comparisons in availability of teaching methods were made using *T* tests, and comparisons in perceived needs for further education using chi-square tests. Correlations were assessed using Spearman’s rank correlation, and Intraclass Correlation Coefficient (ICC) was used to assess between-country consistency in ranking of curriculum topics by preparedness levels. Tests were two-sided and statistical significance was set at *p* < 0.05.

## Results

### Participants

We received responses from 27.2% (2085/7653) of all medical students at 31/34 eligible medical schools in France, and from 26.9% (302/1124) of all medical students at 7/7 eligible medical schools in Sweden (Online Resource 2, Table 1). The majority of respondents in France and Sweden were female (60.4% and 59.5%), and French students were slightly younger on average (24.0 years old vs. 27.2 years old, *p* < 0.001).

**Table 1:** Self-reported preparedness on curriculum topics related to prudent antibiotic use

Topic	France		Sweden	
	Sufficiently prepared		Sufficiently prepared	
	%	(Range)	%	(Range)
To recognise the clinical signs of infection	99	(93–100)	99	(97–100)
To assess the clinical severity of infection (e.g. using criteria, such as the septic shock criteria)	97	(91–100)	96	(92–100)
To interpret biochemical markers of inflammation (e.g. CRP)	97	(83–100)	97	(92–100)
To decide when it is important to take microbiological samples before starting antibiotic therapy	91	(79–97)	95	(92–100)
To use point-of-care tests (e.g. urine dipstick, rapid diagnostic tests for streptococcal pharyngitis)	90	(63–100)	91	(80–97)
To interpret basic microbiological investigations (e.g. blood cultures, antibiotic susceptibility reporting)	88	(73–97)	92	(87–97)
To identify clinical situations when not to prescribe an antibiotic	87	(72–96)	95	(91–98)
To decide the urgency of antibiotic administration in different situations (e.g. < 1 h for severe sepsis, non-urgent for chronic bone infections)	86	(77–93)	85	(73–92)
To assess clinical outcomes and possible reasons for failure of antibiotic treatment	85	(68–100)	81	(73–88)
To prescribe antibiotic therapy according to national/local guidelines	83	(67–91)	92	(80–97)
To differentiate between bacterial and viral upper respiratory tract infections	83	(71–95)	93	(83–100)
To use knowledge of the negative consequences of antibiotic use (bacterial resistance, toxic/adverse effects, cost, *Clostridium difficile* infections)	82	(68–95)	98	(93–100)
To review the need to continue or change antibiotic therapy after 48–72 h, based on clinical evolution and laboratory results	82	(69–91)	81	(73–91)
To differentiate between bacterial colonisation and infection (e.g. asymptomatic bacteriuria)	81	(68–91)	91	(86–94)
To practise effective infection control and hygiene (to prevent spread of bacteria)	78	(63–94)	98	(95–100)
To discuss antibiotic use with patients who are asking for antibiotics, when I feel they are not necessary	76	(60–89)	95	(91–97)
To select initial empirical therapy based on the most likely pathogen(s) and antibiotic resistance patterns, without using guidelines	76	(56–86)	74	(66–80)
To identify indications for combination antibiotic therapy	70	(48–86)	63	(50–73)
To decide when to switch from intravenous (IV) to oral antibiotic therapy	69	(52–86)	75	(70–83)
To assess antibiotic allergies (e.g. differentiating between anaphylaxis and hypersensitivity)	63	(46–77)	76	(64–94)
To use knowledge of the common mechanisms of antibiotic resistance in pathogens	50	(20–81)	86	(78–100)
To measure/audit antibiotic use in a clinical setting, and to interpret the results of such studies	50	(37–63)	61	(51–73)
To work within the multi-disciplinary team in managing antibiotic use in hospitals	49	(36–66)	65	(55–72)
To decide the shortest possible adequate duration of antibiotic therapy for a specific infection	49	(36–70)	59	(45–76)
To prescribe using principles of surgical antibiotic prophylaxis	41	(24–51)	51	(36–63)
To communicate with senior doctors in situations where I feel antibiotics are not necessary, but I feel I am being inappropriately pressured into prescribing antibiotics by senior doctors	38	(21–53)	57	(46–73)
To use knowledge of the epidemiology of bacterial resistance, including local/regional variations	35	(22–59)	79	(70–86)

The table includes results aggregated at medical school level and then at country level. The total number of respondents per question varied for France between *N* = 2065 and 2085, and for Sweden between *N* = 300 and 302

### Global preparedness scores

The country global preparedness score was significantly higher in Sweden (83.4% vs. 74.7%, *p* < 0.001). The medical school global preparedness scores were higher at all seven Swedish institutions (range 81.6–86.1%) than at all 31 French institutions (63.3–81.1%) (Online Resource 2, Table 1).

### Preparedness on individual curriculum topics

Students at Swedish medical schools considered themselves to have higher preparedness on 21 of 27 topics than students at French medical schools (Table 1). The relative ranking order of curriculum topics was consistent between countries (*ρ* = 0.80, *p* < 0.01), although there were a few topics in which there were large differences. For example, Swedish students felt far more prepared to practise effective infection control and hygiene (ranked 2nd vs. 15th), to discuss antibiotic use with patients who are asking for antibiotics, when the student feels they are not necessary (7th vs. 16th), and to use knowledge of the epidemiology of bacterial resistance, including local/regional variations (18th vs. 27th); French students felt more prepared to use point-of-care tests (5th vs. 12th), to decide the urgency of antibiotic administration in different situations (8th vs. 15th), and to assess clinical outcomes and possible reasons for failure of antibiotic treatment (9th vs. 16th).

The three topics with the highest reports of not having teaching were the same in both countries: (a) to communicate with senior doctors in situations where the student feels antibiotics are not necessary, but they feel they are being inappropriately pressured into prescribing antibiotics by senior doctors (9.6% of students in France reporting no teaching vs. 3.4% in Sweden); (b) to work within the multi-disciplinary team in managing antibiotic use in hospitals (8.1% vs. 3.7%); (c) to measure/audit antibiotic use in a clinical setting, and to interpret the results of such studies (5.5% vs. 3.0%). These three topics were also in the bottom six topics by preparedness level in both countries.

### Availability and usefulness of teaching methods

With the exception of peer or near-peer teaching, all teaching methods were reported to have higher availability at institutions in Sweden than in France (Table 2). There was a strong positive correlation in both countries between the availability of a teaching method and its perceived usefulness (France: *ρ* = 0.85, *p* < 0.01; Sweden *ρ* = 0.90, *p* < 0.01). No strong correlations were observed between availability of individual teaching methods at medical schools and the medical school global preparedness score.

**Table 2:** Perceived availability and usefulness of teaching methods on antibiotic use

Teaching method	France			Sweden		
	Useful or very useful		Not available	Useful or very useful		Not available
	%	(Range)	%	%	(Range)	%
Discussions of clinical cases and vignettes	82	(64–93)	11	92	(86–100)	1^**^
Peer or near-peer teaching	76	(60–93)	25	72	(60–88)	41^**^
Infectious diseases clinical placements	71	(43–94)	31	92	(84–97)	2^**^
Small group teaching (< 15 people)	68	(37–90)	46	94	(88–100)	9^**^
Lectures (≥ 15 people)	62	(32–79)	16	90	(82–100)	0^**^
Active learning assignments	53	(22–81)	45	56	(41–70)	20^**^
Microbiology clinical placements	45	(20–70)	48	49	(25–70)	41
E-learning	44	(14–67)	57	37	(17–54)	54
Role play or communication skills sessions	39	(8–67)	67	44	(0–78)	49

^**^Comparison in unavailability of a teaching method between medical schools in Sweden and France is significant at *p* < 0.001

### Need for more education

**Fig. 1:**
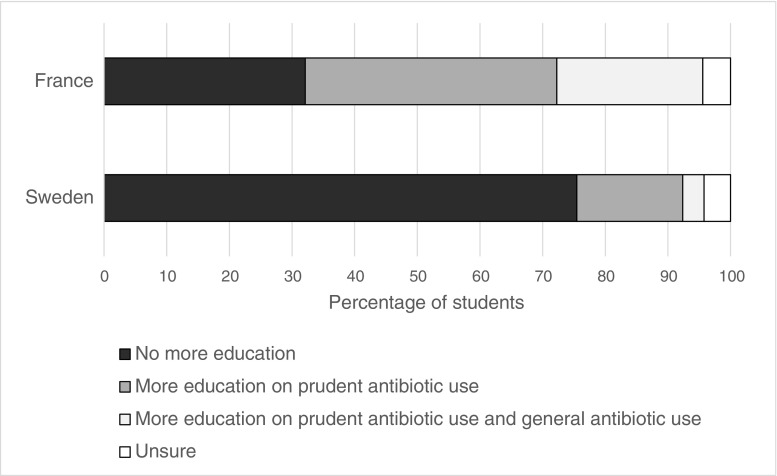
Self-reported need for more education on antibiotic use

Students from French medical schools were more likely to report needing more education on antibiotic use for their work as junior doctors (63.5% vs. 20.3%, *p* < 0.001, Fig. 1). The self-reported needs for more education varied widely at French medical schools (37.0–83.3%), and were strongly inversely correlated with medical school global preparedness scores (*ρ* = − 0.77, *p* < 0.001). There was less variation at Swedish medical schools (13.3–35.9%), and a weaker correlation with medical school global preparedness scores (*ρ* = − 0.37, *p* < 0.001).

## Discussion

### Overall preparedness and needs for further education

We assessed self-reported preparedness on topics related to responsible antibiotic use among a large number of final year students at medical schools in Sweden and France. Students at Swedish medical schools felt more prepared on most topics, and remarkably, overall preparedness levels were higher at all seven Swedish schools than the 31 French schools.

There are several potential explanations for these results. First, students may have reached similar absolute levels of preparedness in both countries, but could vary in their perceptions of the preparedness levels needed for their work. The management of infections in France may be viewed by students as more ‘complex’ than in Sweden: there is a higher prevalence of multi-drug-resistant bacteria [7]; a higher number of antibiotics are available and recommended in guidelines and textbooks, but guidelines are quite complicated and are used less consistently [14]; students almost exclusively rotate through specialised tertiary-care hospitals, so they are exposed to complicated patients and disease management. Furthermore, students may perceive that higher levels of preparedness are required if they enter directly into specialty training (France), than into an internship (Sweden). A second explanation is that cultural factors may lead students in France to self-evaluate themselves as being less prepared. For example, Deschepper et al report that Swedish culture is associated with a higher level of tolerance for uncertainty and ambiguity than French culture [15], and a systematic review suggested that low self-reported preparedness levels for working as a junior doctor are linked with certain factors including ‘uncertainty avoidance’ [16].

An alternative hypothesis is that students in Sweden are actually more prepared. This is supported by the far higher levels of self-reported need for further education among students at French medical schools, and a correlation at individual schools between overall preparedness levels and need for further education. Undergraduate training duration is similar in both countries; however, there may be key differences in the teaching and learning opportunities that students encounter. Eight of the nine teaching methods had higher availability in Sweden; students’ learning may have been enhanced through exposure to the same content presented in different modalities. Studies investigating preparedness among junior doctors in the UK [17] and medical students in the Netherlands [18] suggest that preparedness for prescribing in practice is best achieved by supervised clinical exposure and that this cannot be easily substituted by other forms of learning. It is striking that nearly a third of medical students in France reported not undertaking a clinical placement in infectious diseases. In Sweden, it is common for students to undertake paid work in hospitals during their holidays as ‘physician assistants’; these positions of responsibility may provide a qualitatively different form of clinical decision-making experience, albeit without the legal ability to sign prescriptions. If students in Sweden are truly more prepared to prescribe antibiotics prudently, then an intriguing possibility is that this has translated over time into more responsible antibiotic use, and in turn led to lower prevalence of multi-drug resistant bacteria. Robust studies assessing the relationship between self-reported preparedness and actual practices are needed to investigate this further.

### Variations in preparedness on individual topics

For some topics, there were wide differences in the relative ranking of preparedness between students in France and Sweden (but not necessarily differences in absolute preparedness levels), such as practising effective infection control and hygiene. These disparities might reflect differences in national priorities, which translate into consistent emphases placed on certain topics at individual medical schools. Previous studies among medical students [12] and junior doctors [19] in France found that over a quarter of respondents did not identify hand hygiene as an important contributor to antimicrobial resistance. Other differences may be due to the availability and/or endorsement of tools at a national level (students in France felt relatively more prepared to use point-of-care tests), as well as due to consistent differences in how teaching is delivered (for example, the department responsible for training on different topics).

### Methodological considerations

The response rate was 27% in both Sweden and France; however, it is comparable to that of other similar studies on this topic [12, 20], and we do not believe it will have introduced significant selection bias given the topic of the study and the lack of incentives to participate. Furthermore, since students participated from all medical schools in Sweden and almost all in France, the results are likely to be highly relevant to all institutions in these countries, and potentially even to postgraduate training programmes. A further limitation is that we did not include any objective assessment of preparedness, in part, because no validated set of case vignettes exists for assessing preparedness on antibiotic use.

### Further work

An appropriate next step is to systematically evaluate the formal curricula, teaching, and assessment methods used in Sweden and France, to identify how to address potential weaknesses in knowledge and skills. This can be supported by comparing curricula with internationally agreed upon sets of competencies in antimicrobial stewardship and prescribing [3, 21], and complemented by qualitative studies among medical students and faculty. We recommend that medical schools with high and low levels of preparedness share experiences with the aims of improving student learning. In a time of growing global antimicrobial resistance, strong efforts should be made to ensure that all students leave medical school feeling prepared to prescribe antimicrobials responsibly.

## Electronic supplementary material

ESM 1 (PDF 916 kb)

ESM 2 (PDF 52 kb)

## Data Availability

The datasets generated during and/or analysed during the current study are available from the corresponding author on reasonable request.

## References

[1] Pulcini C, Gyssens IC (2013). How to educate prescribers in antimicrobial stewardship practices. Virulence.

[2] Ohl CA, Luther VP (2014). Health care provider education as a tool to enhance antibiotic stewardship practices. Infect Dis Clin N Am.

[3] World Health Organisation (2018) WHO competency framework for health workers’ education and training on antimicrobial resistance. http://apps.who.int/medicinedocs/documents/s23443en/s23443en.pdf. Accessed 26 November 2018

[4] World Health Organisation (2015) Global action plan on antimicrobial resistance. http://apps.who.int/gb/ebwha/pdf_files/WHA68/A68_R7-en.pdf?ua=1. Accessed 26 November 2018

[5] Dyar OJ, Nathwani D, Monnet DL, Gyssens IC, Stålsby Lundborg C (2018). Do medical students feel prepared to prescribe antibiotics responsibly? Results from a cross-sectional survey in 29 European countries. J Antimicrob Chemother.

[6] Ferech M, Coenen S, Malhotra-Kumar S, Dvorakova K, Hendrickx E, Suetens C (2006). European Surveillance of Antimicrobial Consumption (ESAC): outpatient antibiotic use in Europe. J Antimicrob Chemother.

[7] European Centre for Disease Prevention and Control (2015) Antimicrobial resistance surveillance in Europe 2014. Annual report of the European Antimicrobial Resistance Surveillance Network (EARS-Net). https://ecdc.europa.eu/sites/portal/files/media/en/publications/Publications/antimicrobial-resistance-europe-2014.pdf. Accessed 26 Nov 2018

[8] Pulcini C, ESGAP AMOXDOSE working group (2017). Amoxicillin dosing recommendations are very different in European countries: a cross-sectional survey. Clin Microbiol Infect.

[9] Segouin C, Jouquan J, Hodges B, Bréchat P-H, David S, Maillard D (2007). Country report: medical education in France. Med Educ.

[10] Lindgren S, Brännström T, Hanse E, Ledin T, Nilsson G, Sandler S (2011). Medical education in Sweden. Med Teach.

[11] Pulcini C, Wencker F, Frimodt-Møller N, Kern WV, Nathwani D, Rodríguez-Baño J (2015). European survey on principles of prudent antibiotic prescribing teaching in undergraduate students. Clin Microbiol Infect.

[12] Dyar OJ, Pulcini C, Howard P, Nathwani D, Beovic B, Harbarth S (2014). European medical students: a first multicentre study of knowledge, attitudes and perceptions of antibiotic prescribing and antibiotic resistance. J Antimicrob Chemother.

[13] Abbo LM, Cosgrove SE, Pottinger PS, Pereyra M, Sinkowitz-Cochran R, Srinivasan A (2013). Medical students’ perceptions and knowledge about antimicrobial stewardship: how are we educating our future prescribers?. Clin Infect Dis.

[14] Monnet DL, Ferech M, Frimodt-Møller N, Goossens H (2005). The more antibacterial trade names, the more consumption of antibacterials: a European study. Clin Infect Dis.

[15] Deschepper R, Grigoryan L, Lundborg CS, Hofstede G, Cohen J, Kelen G, Der V (2008). Are cultural dimensions relevant for explaining cross-national differences in antibiotic use in Europe?. BMC Health Serv Res.

[16] Cameron A, Millar J, Szmidt N, Hanlon K, Cleland J (2014). Can new doctors be prepared for practice? A review. Clin Teach.

[17] Rothwell C, Burford B, Morrison J, Morrow G, Allen M, Davies C (2012). Junior doctors prescribing: enhancing their learning in practice. Br J Clin Pharmacol.

[18] Brinkman DJ, Tichelaar J, van Agtmael MA, Schotsman R, de Vries TPGM, Richir MC (2014). The prescribing performance and confidence of final-year medical students. Clin Pharmacol Ther.

[19] Pulcini C, Williams F, Molinari N, Davey P, Nathwani D (2011). Junior doctors’ knowledge and perceptions of antibiotic resistance and prescribing: a survey in France and Scotland. Clin Microbiol Infect.

[20] Minen MT, Duquaine D, Marx MA, Weiss D (2010). A survey of knowledge, attitudes, and beliefs of medical students concerning antimicrobial use and resistance. Microb Drug Resist.

[21] Dyar OJ, Beović B, Pulcini C, Tacconelli E, Hulscher M, Cookson B (2019). ESCMID generic competencies in antimicrobial prescribing and stewardship: towards a European consensus. Clin Microbiol Infect 2018.

