# Supplementary material for: Preparedness to prescribe antibiotics responsibly: a comparison between final year medical students in France and Sweden
Source: Eur J Clin Microbiol Infect Dis. 2019 Feb 15;38(4):711–7. doi: 10.1007/s10096-019-03494-2 (PMC6425071; doi:10.1007/s10096-019-03494-2)
Supplement: Supplementary file 1 — (PDF 916 kb) [file 10096_2019_3494_MOESM1_ESM.pdf]

Online resource 1:

Preparedness to prescribe antibiotics responsibly: a comparison between final year medical students in France and Sweden

+Oliver James DYAR, Maria LUND, Cecilia LINDSJÖ, Cecilia STÅLSBY LUNDBORG, Céline PULCINI, on behalf of the French-Swedish Student-PREPARE ESGAP working group

+Corresponding author: Oliver Dyar, Department of Public Health Sciences, Karolinska Institutet, Stockholm, Sweden. Email: oliver.dyar@ki.se

Student-PREPARE 2015

Survey introduction and demographic questions

The purpose of this study is to learn how medical students in Europe are taught about antibiotic prescribing and using antibiotics appropriately ('prudent antibiotic prescribing'). We greatly appreciate your completion of the survey. The results will be used to suggest improvements to existing curricula, and will be published in national and international peer-reviewed journals.

The survey should take five to ten minutes to complete, please try to answer all questions. All responses are anonymous and will be stored confidentially.

1. How old are you? (in years)

[ ]

2. What is your sex?

○ Female

○ Male

3. Are you a citizen in the country in which you are studying medicine?

○ Yes

○ No (please write the country you are a citizen in)

[ ]

4. Will you complete your medical studies before the end of February 2016?

- ◯ Yes
- ◯ No

Other (please specify)

Please note, this survey is only for students who are in their final year (or in their final two semesters)

5. What is the full duration of your medical training in your medical school?

- ◯ 4 years
- ◯ 5.5 years / 11 semesters
- ◯ 7 years
- ◯ 5 years
- ◯ 6 years

Other (please specify)

6. Which medical school are you studying at? (medical schools are listed under each country, in alphabetic order)

Other (please specify)

Curriculum content: principles for prudent antibiotic use

Please note that this is the longest section of the survey.

This section asks how well you feel your studies at medical school have prepared you for your practice as a junior doctor on the following topics of prudent antibiotic use (including all courses, lectures, clinical placements, training at the bedside, exams, and self-directed learning).

For each question, please respond "I feel... (1 = not at all prepared, 4 = sufficiently prepared, 7 = very well prepared) to...".

If there are questions where you have not had any teaching, please select the 'no teaching provided', irrespective of how well prepared you feel on this topic.

7. Diagnosis of infection

I feel...

	No teaching was provided	1 (Not at all prepared)	2	3	4 (Sufficiently prepared)	5	6	7 (Very well prepared)	I am unsure how I feel	I do not understand the question
To recognise the clinical signs of infection	◯	◯	◯	◯	◯	◯	◯	◯	◯	◯
To assess the clinical severity of infection (e.g using criteria, such as the septic shock criteria)	◯	◯	◯	◯	◯	◯	◯	◯	◯	◯
To use point-of-care tests (e.g. urine dipstick, rapid diagnostic tests for streptococcal pharyngitis)	◯	◯	◯	◯	◯	◯	◯	◯	◯	◯
To interpret biochemical markers of inflammation (e.g. CRP)	◯	◯	◯	◯	◯	◯	◯	◯	◯	◯
To decide when it is important to take microbiological samples before starting antibiotic therapy	◯	◯	◯	◯	◯	◯	◯	◯	◯	◯
To interpret basic microbiological investigations (e.g. blood cultures, antibiotic susceptibility reporting)	◯	◯	◯	◯	◯	◯	◯	◯	◯	◯

8. Indications for no antibiotic treatment

I feel...

	No teaching was provided	1 (Not at all prepared)	2	3	4 (Sufficiently prepared)	5	6	7 (Very well prepared)	I am unsure how I feel	I do not understand the question
To identify clinical situations when not to prescribe an antibiotic										
To differentiate between bacterial colonisation and infection (e.g. asymptomatic bacteriuria)										
To differentiate between bacterial and viral upper respiratory tract infections										

9. Initial antibiotic therapy

I feel...

	No teaching was provided	1 (Not at all prepared)	2	3	4 (Sufficiently prepared)	5	6	7 (Very well prepared)	I am unsure how I feel	I do not understand the question
To select initial empirical therapy based on the most likely pathogen(s) and antibiotic resistance patterns, without using guidelines										
To decide the urgency of antibiotic administration in different situations (e.g <1 hr for severe sepsis, non-urgent for chronic bone infections)										

	No teaching was provided	1 (Not at all prepared)	2	3	4 (Sufficiently prepared)	5	6	7 (Very well prepared)	I am unsure how I feel	I do not understand the question
To prescribe antibiotic therapy according to national/local guidelines	◯	◯	◯	◯	◯	◯	◯	◯	◯	◯
To assess antibiotic allergies (e.g. differentiating between anaphylaxis and hypersensitivity)	◯	◯	◯	◯	◯	◯	◯	◯	◯	◯
To identify indications for combination antibiotic therapy	◯	◯	◯	◯	◯	◯	◯	◯	◯	◯
To decide the shortest possible adequate duration of antibiotic therapy for a specific infection	◯	◯	◯	◯	◯	◯	◯	◯	◯	◯
To prescribe using principles of surgical antibiotic prophylaxis	◯	◯	◯	◯	◯	◯	◯	◯	◯	◯

10. Reassessment of antibiotic therapy

I feel...

	No teaching was provided	1 (Not at all prepared)	2	3	4 (Sufficiently prepared)	5	6	7 (Very well prepared)	I am unsure how I feel	I do not understand the question
To review the need to continue or change antibiotic therapy after 48-72 hours, based on clinical evolution and laboratory results										
To assess clinical outcomes and possible reasons for failure of antibiotic treatment										
To decide when to switch from intravenous (IV) to oral antibiotic therapy										

11. Quality of care

I feel...

	No teaching was provided	1 (Not at all prepared)	2	3	4 (Sufficiently prepared)	5	6	7 (Very well prepared)	I am unsure how I feel	I do not understand the question
To measure/audit antibiotic use in a clinical setting, and to interpret the results of such studies										
To work within the multi-disciplinary team in managing antibiotic use in hospitals										

12. Communication skills

I feel...

	No teaching was provided	1 (Not at all prepared)	2	3	4 (Sufficiently prepared)	5	6	7 (Very well prepared)	I am unsure how I feel	I do not understand the question
To discuss antibiotic use with patients who are asking for antibiotics, when I feel they are not necessary	◯	◯	◯	◯	◯	◯	◯	◯	◯	◯
To communicate with senior doctors in situations where I feel antibiotics are not necessary, but I feel I am being inappropriately pressured into prescribing antibiotics by senior doctors	◯	◯	◯	◯	◯	◯	◯	◯	◯	◯

13. Antibiotic resistance

I feel...

	No teaching was provided	1 (Not at all prepared)	2	3	4 (Sufficiently prepared)	5	6	7 (Very well prepared)	I am unsure how I feel	I do not understand the question
To use knowledge of the common mechanisms of antibiotic resistance in pathogens	◯	◯	◯	◯	◯	◯	◯	◯	◯	◯
To use knowledge of the epidemiology of bacterial resistance, including local/regional variations	◯	◯	◯	◯	◯	◯	◯	◯	◯	◯
To practise effective Infection control and hygiene (to prevent spread of bacteria)	◯	◯	◯	◯	◯	◯	◯	◯	◯	◯
To use knowledge of the negative consequences of antibiotic use (bacterial resistance, toxic/adverse effects, cost, *Clostridium difficile* infections)	◯	◯	◯	◯	◯	◯	◯	◯	◯	◯

Teaching methods used for antibiotic education

14. Which of the following methods have been used at your medical school for teaching about prudent antibiotic use (the topics from the previous set of questions), and how useful were they?

	Teaching method was not used	Not at all useful	Neutral	Useful	Very useful	I am unsure	I do not understand the question
Lectures (with > 15 people)	○	○	○	○	○	○	○
Small group teaching (with <15 people)	○	○	○	○	○	○	○
Discussions of clinical cases and vignettes	○	○	○	○	○	○	○
Active learning assignments (e.g. article reading, group work, preparing an oral presentation)	○	○	○	○	○	○	○
E-learning	○	○	○	○	○	○	○
Role play or communication skills sessions dealing with patients demanding antibiotic therapy	○	○	○	○	○	○	○
Infectious diseases clinical placement (i.e. clinical rotation or training in infectious diseases, involving patients)	○	○	○	○	○	○	○
Microbiology clinical placement	○	○	○	○	○	○	○

	Teaching method was not used	Not at all useful	Neutral	Useful	Very useful	I am unsure	I do not understand the question
Peer or near-peer teaching (i.e. teaching led by other students, or recently qualified doctors)	○	○	○	○	○	○	○

Others used often (please specify)

15. Overall, do you feel you have received sufficient teaching at medical school in antibiotic use for your future practice as a junior doctor?

- ○ Yes
- ○ No, I feel I had enough teaching on general antibiotic treatment, but I need more on prudent antibiotic use
- ○ No, I feel I need more education on both general antibiotic treatment and prudent antibiotic use
- ○ I am unsure

Other (please specify)

16. Have any of your medical school examinations included questions on antibiotic treatment?

- ○ Yes
- ○ No
- ○ Unsure

Other (please specify)

17. How do you think teaching on antibiotic treatment and prudent antibiotic use can be improved?

18. How did you find the language used in this study?

- ◯ I could understand everything or almost everything
- ◯ I could understand most questions, it was alright for the survey to be in English
- ◯ Many questions were difficult to understand, I would have preferred the survey in my native language
- ◯ Most of the survey was difficult to understand, I would have preferred the survey in my native language

Other (please specify)
