# Supplementary material for: Preparedness to prescribe antibiotics responsibly: a comparison between final year medical students in France and Sweden
Source: Eur J Clin Microbiol Infect Dis. 2019 Feb 15;38(4):711–7. doi: 10.1007/s10096-019-03494-2 (PMC6425071; doi:10.1007/s10096-019-03494-2)
Supplement: Supplementary file 2 — (PDF 52 kb) [file 10096_2019_3494_MOESM2_ESM.pdf]

Online resource 2:

Preparedness to prescribe antibiotics responsibly: a comparison between final year medical students in France and Sweden

+Oliver James DYAR, Maria LUND, Cecilia LINDSJÖ, Cecilia STÅLSBY LUNDBORG, Céline PULCINI, on behalf of the French-Swedish Student-PREPARE ESGAP working group

+Corresponding author: Oliver Dyar, Department of Public Health Sciences, Karolinska Institutet, Stockholm, Sweden. Email: oliver.dyar@ki.se

Table 1. Numbers of participating students and global preparedness scores for medical schools

Country	Medical school	No. of participating students	Global preparedness score (%)
France	Amiens	52	69.8
	Angers	75	71.6
	Besançon	60	74.0
	Bordeaux	140	76.0
	Brest	24	72.3
	Caen	54	77.8
	Dijon	37	79.4
	Grenoble	84	81.1
	Lille	84	75.9
	Limoges	47	68.5
	Lyon	148	72.3
	Marseille	64	63.3
	Montpellier	54	76.0
	Nancy	107	73.6
	Nantes	60	75.2
	Nice	35	77.1
	Nîmes	22	77.2
	Paris 5 – Université Paris Descartes	123	80.9
	Paris 6 – Université Pierre et Marie Curie	105	78.5
	Paris 7	111	77.4
	Paris-Sud - Université Paris 11	33	77.5
	Paris 13	38	75.4
	Poitiers	63	74.4
	Reims	57	72.9
	Rennes	74	71.4
	Rouen	54	74.6
	Saint-Etienne	44	69.8
	Strasbourg	41	75.4
	Toulouse	122	78.6
	Tours	36	70.9
	Versailles (UFR des sciences de la santé Simone Veil)	37	76.8
Sweden	Gothenburg University	50	81.6
	Karolinska Institutet	69	84.0
	Linköping University	58	84.0
	Lund University	36	81.8
	Umeå University	39	82.3
	Uppsala University Faculty of Medicine	35	86.1
	Örebro University	15	84.3

Higher global preparedness scores reflect students feeling prepared across a greater proportion of topics, and ranged from 63.3% to 81.1% in France, and from 81.6% to 86.1% in Sweden.
